# Supplementary material for: Stem Cell-Secreted Allogeneic Elastin-Rich Matrix with Subsequent Decellularization for the Treatment of Critical Valve Diseases in the Young
Source: Bioengineering (Basel). 2022 Oct 20;9(10):587. doi: 10.3390/bioengineering9100587 (PMC9598163; doi:10.3390/bioengineering9100587)
Supplement: Supplementary file 1 [file bioengineering-09-00587-s001.zip › bioengineering-1927043-supplementary.pdf]

Supplementary Data

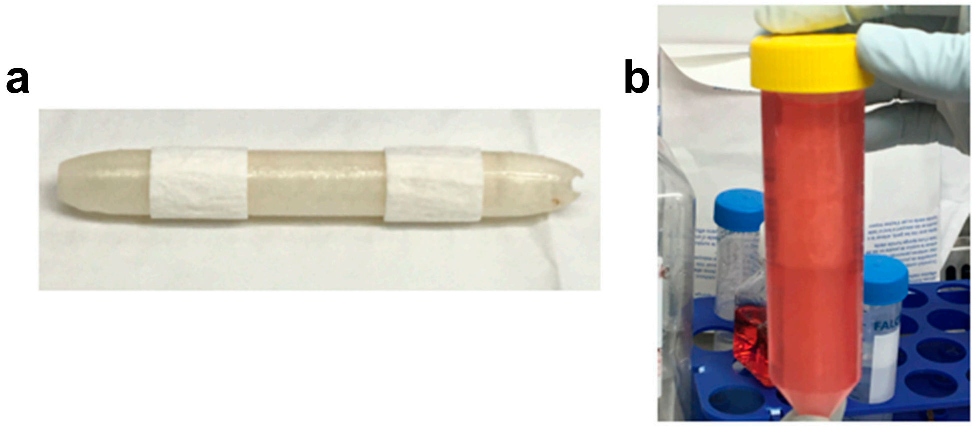

Figure S1. Torpedo for hBMMSC cell seeding of PSIS valve. (**a**) Two valves placed on the torpedo. (**b**) Valves transferred to a vented conical tube for 8 days in rotisserie.

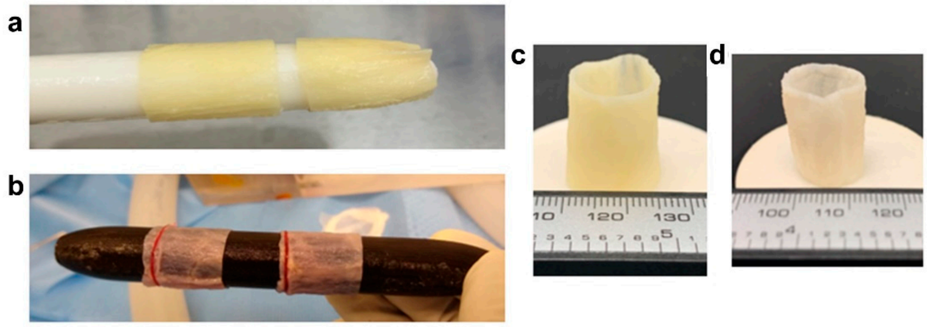

Figure S2. PSIS valves post static and dynamic culture. (**a**) Two PSIS valves on the torpedo holder, seeded with MSCs for 8 days in rotisserie and kept in rotisserie for 14 days thereafter (static group). (**b**) Two PSIS valves on the torpedo holder, seeded with hBMMSCs for 8 days and then loaded into the torpedo bioreactor for 14 days. (**c**) Static and (**d**) dynamic PSIS valves, respectively, removed from the torpedo holder after 22 days.

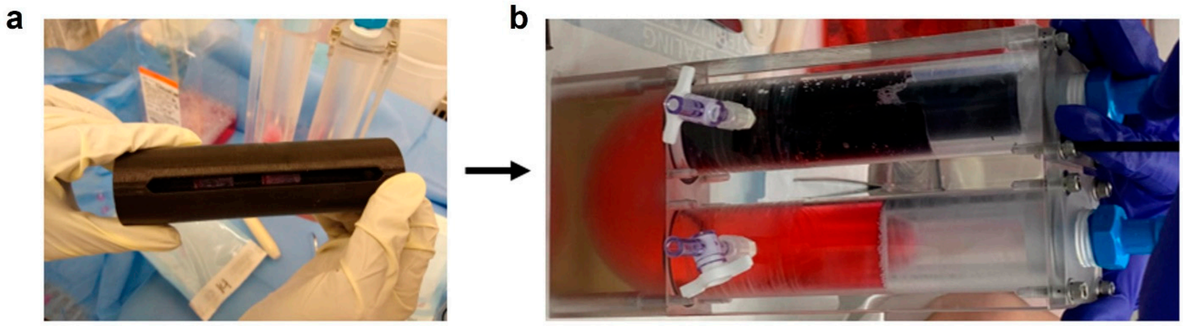

Figure S3. Loaded "torpedo" bioreactor. (**a**) Two PSIS valves on the torpedo holder, seeded with hBMMSCs for 8 days in rotisserie, are placed in the outer housing. (**B**) These PSIS valves are then loaded into the torpedo bioreactor for 14 days.

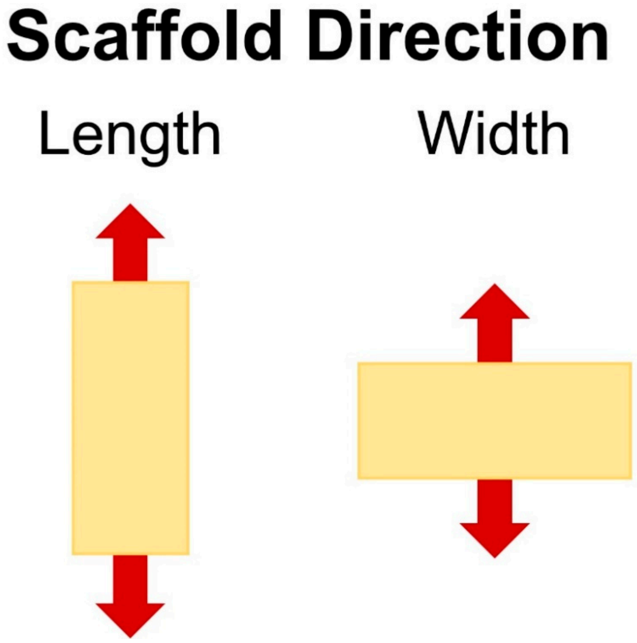

Figure S4. Scaffold direction for uniaxial tensile tests. Red arrows represent the direction in which the scaffold is stretched.

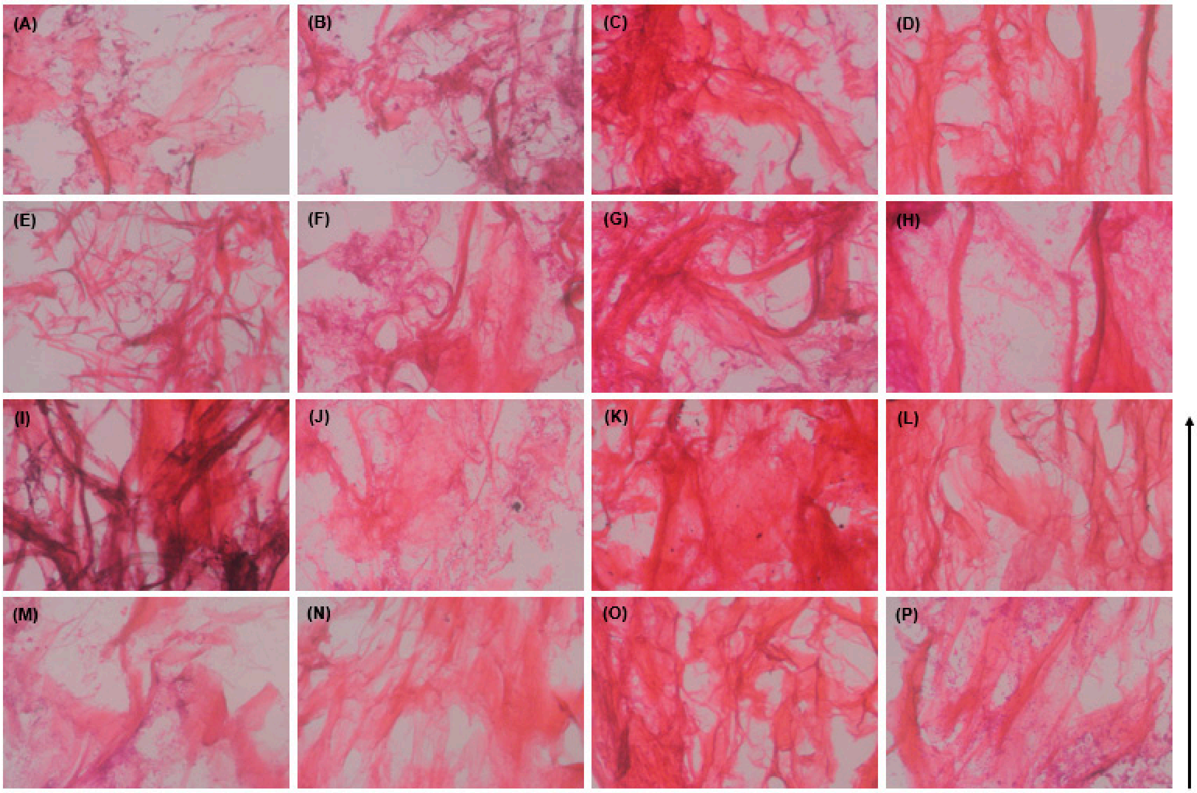

Figure S5. Morphology and cellular infiltration of PSIS valves. (**A-H**) Static and (**I-P**) dynamic PSIS valves were morphologically assessed via H&E staining. Both groups had cellular infiltration and tissue formation. The PSIS valves

are denser in tissue as the depth increases from (**A, E, I, M**) 80 µm, (**B, F, J, N**) 176 µm, (**C, G, K, O**) 272 µm to (**D, H, L, P**) 368 µm. The (**A-H**) static group is scattered in orientation, while the (**I-P**) dynamic group oriented towards the direction of flow. The arrow signifies the direction of flow. Magnification 50x.

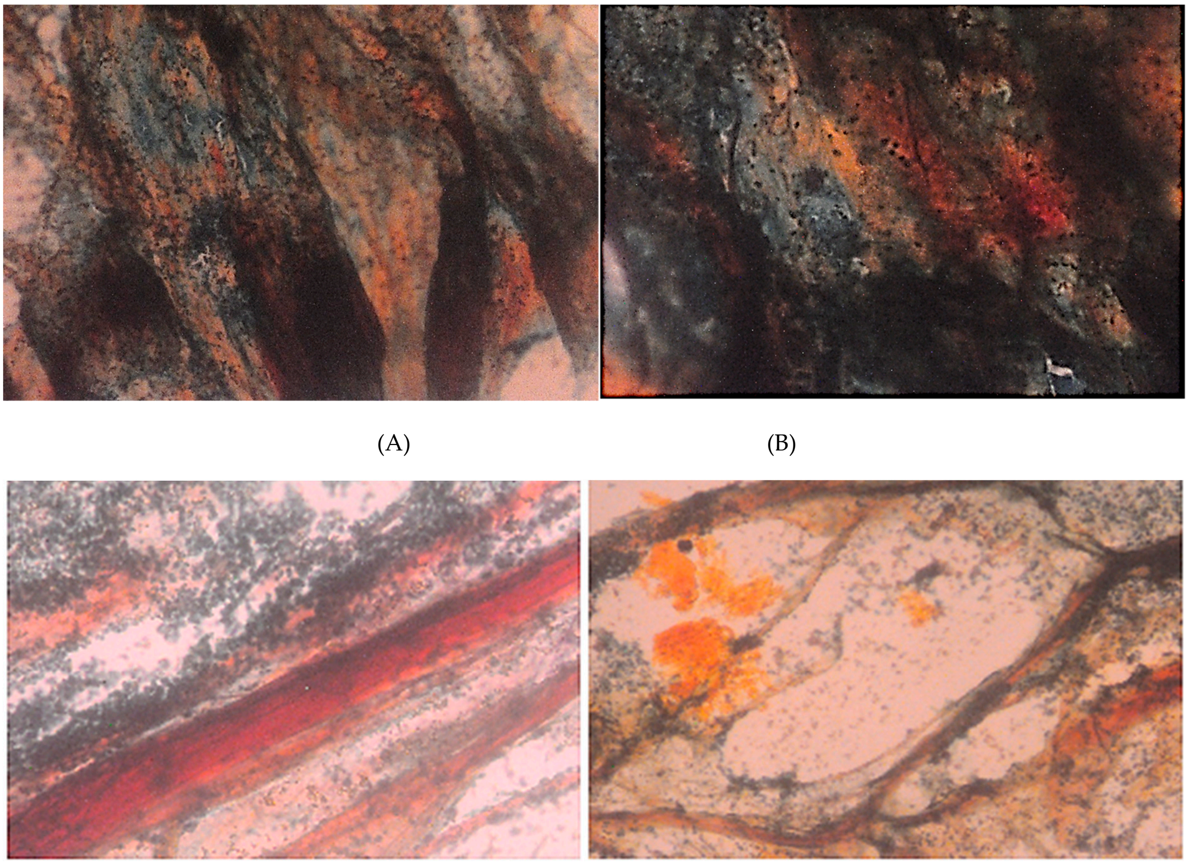

(A) (B)

(C) (D)

Figure S6: An additional torpedo bioreactor run confirming that the 0.2 OSI mechanical conditioning promoted the production of elastin in the ECM secreted by the human bone marrow-derived mesenchymal stem cells (hBMMSCs): (A) and (B) - MOVAT Pentachromic stained hBMMSCs-seeded Engineered ECM valves (n=2) after 8 days of static rotisserie culture followed by an additional 14 days at 0.2 OSI dynamic culture. (C) and (D) - MOVAT Pentachromic stained hBMMSCs-seeded Engineered ECM valves (n=2) after 22 days of static rotisserie culture. Black denotes elastin, gray/black is hBMMSCs nuclei, red is fibrin and green is mucins.

Table S1. Average % ± SEM of unfilled *de novo* ECM on *in vitro* PSIS tubular valves. Various images throughout the depth of the samples were assessed to determine the average percentages ± SEM of unfilled *de novo* ECM on the PSIS tubular constructs. A significant difference (p<0.05) was found between the two groups. * Signifies a significant difference.

PSIS Valve Group	% Unfilled Tissue Formation
"Static" Control	53% ± 2.84*
Dynamic	39% ± 1.83*

Table S2. Average ± SEM of the linear stiffness and maximum tensile stress of the control and TX-100 scaffolds (n=6/group; n=3/direction).

Treatment	Direction	Linear Stiffness (MPa)	Max Tensile Stress (MPa)
Control	Length	16.14 ± 1.79	2.72 ± 0.32
	Width	9.65 ± 0.82	1.67 ± 0.00
TX-100	Length	21.09 ± 3.05	2.98 ± 0.13
	Width	9.59 ± 1.25	2.40 ± 0.19

Table S3. Results of statistical analysis on uniaxial tensile tests. A t-test was performed in this statistical analysis. It was found that there was no significant difference between treatments regarding linear stiffness and maximum tensile stress in either direction.

Comparison Group	*t*-Test p-value
Width Linear Stiffness	0.97 (>0.05)
Length Linear Stiffness	0.26 (>0.05)
Width Max Stress	0.07 (>0.05)
Length Max Stress	0.49 (>0.05)
